# Compton Camera X-Ray Fluorescence Imaging Design and Image Reconstruction Algorithm Optimization

**DOI:** 10.3390/jimaging11090300

**Published:** 2025-09-03

**Authors:** Shunmei Lu, Kexin Peng, Peng Feng, Cheng Lin, Qingqing Geng, Junrui Zhang

**Affiliations:** 1 College of Nuclear Technology and Automation Engineering, Chengdu University of Technology, Chengdu 610059, China; lsm@stu.cdut.edu.cn (S.L.); 2022050512@stu.cdut.edu.cn (C.L.); gengqing@stu.cdut.edu.cn (Q.G.); 2024020579@stu.cdut.edu.cn (J.Z.); 2 The Key Laboratory of Optoelectronic Technology and Systems, Ministry of Education, Chongqing University, Chongqing 400044, China; coe-fp@cqu.edu.cn

**Keywords:** Compton camera, X-ray fluorescence, image reconstruction, MLEM, three-dimensional reconstruction

## Abstract

Traditional X-ray fluorescence computed tomography (XFCT) suffers from issues such as low photon collection efficiency, slow data acquisition, severe noise interference, and poor imaging quality due to the limitations of mechanical collimation. This study proposes to design an X-ray fluorescence imaging system based on bilateral Compton cameras and to develop an optimized reconstruction algorithm to achieve high-quality 2D/3D imaging of low-concentration samples (0.2% gold nanoparticles). A system equipped with bilateral Compton cameras was designed, replacing mechanical collimation with “electronic collimation”. The traditional LM-MLEM algorithm was optimized through improvements in data preprocessing, system matrix construction, iterative processes, and post-processing, integrating methods such as Total Variation (TV) regularization (anisotropic TV included), filtering, wavelet-domain constraints, and isosurface rendering. Successful 2D and 3D reconstruction of 0.2% gold nanoparticles was achieved. Compared with traditional algorithms, improvements were observed in convergence, stability, speed, quality, and accuracy. The system exhibited high detection efficiency, angular resolution, and energy resolution. The Compton camera-based XFCT overcomes the limitations of traditional methods; the optimized algorithm enables low-noise imaging at ultra-low concentrations and has potential applications in early cancer diagnosis and material analysis.

## 1. Introduction

X-ray fluorescence computed tomography (XFCT) technology determines elemental composition based on the specific energy fluorescence spectra emitted by different elements, offering advantages such as non-destructiveness, high spatial resolution, and capabilities for quantitative and qualitative analysis [1,2]. However, this technology relies on mechanical collimators, which restrict photon paths by physical occlusion, resulting in reduced photon absorption efficiency and necessitating multiple scans from different angles, thereby increasing radiation exposure risk. Furthermore, existing image reconstruction algorithms face challenges including long data acquisition times, high computational complexity, ineffective noise removal, and poor imaging accuracy, significantly limiting the application and development of XFCT technology.

Currently, researchers have achieved progress in XFCT technology research [3,4]. Hardware-wise, the XFCT system is continuously being optimized through improvements in mechanical collimator design [5], radiation sources [5], and detector factors to enhance X-ray absorption and sensitivity [6,7]. However, much of this work targets specific elements or sample materials, and overall system performance remains relatively low. Algorithmically, compared to common methods such as Simple Back Projection (SBP) [8], Filtered Back Projection (FBP) [9], and the Algebraic Reconstruction Technique (ART) [10], the Maximum Likelihood Expectation Maximization (MLEM) algorithm demonstrates superior performance in noise resistance, tolerance to incomplete data, and imaging resolution, making it the most commonly used reconstruction algorithm. Liu et al. demonstrated the feasibility and effectiveness of the MLEM algorithm for L-shell XFCT image reconstruction, achieving a relative error below 14.1%. However, the use of X-ray sources with varying energies in that study resulted in high radiation doses to samples, and noise interference was not effectively suppressed, limiting its practical application [11]. Subsequently, some researchers proposed multi-color X-ray fluorescence CT imaging based on sheet-beam geometry, using the MLEM algorithm under a discretization model to achieve accurate reconstruction. However, this approach was limited to two-dimensional reconstruction and did not explore three-dimensional imaging [12].

The Compton Camera (CC) eliminates the need for mechanical collimation by utilizing the Compton scattering principle to achieve “electronic collimation” for photon direction tracking [13]. Due to its advantages of compact size, light weight, high sensitivity, high energy resolution, and multi-energy imaging capability—enabling three-dimensional imaging from a single scan—it has garnered significant attention in XFCT imaging systems in recent years. Research primarily focuses on theoretical simulation, feasibility studies, imaging system development, and algorithm development. In 2016, Vernekohl et al. verified the feasibility of unilateral Compton cameras in human lung imaging through Monte Carlo simulations. Specifically, they performed imaging targeting fluorescent photons generated by gold nanoparticles, achieving an angular resolution of approximately 12.5°. However, the system was limited by single-angle detection (with a coverage range ≤ 120°). For samples with low concentrations (<0.5% *w*/*v*), the number of effective events was relatively low, which easily led to blurred imaging [14,15]. In 2021, a research team constructed an X-ray fluorescence imaging experimental platform based on a Compton camera using a Si-Timepix3 single-layer detector for the first time. This study verified the feasibility and imaging capability of the single-layer Timepix3 detector in Compton camera-based X-ray imaging systems. Nevertheless, there remained significant room for improvement in aspects such as spatial resolution, event selection efficiency, detection sensitivity, and noise suppression, as well as clinical translation [16]. In 2022, the same team successfully imaged a 100 mg/mL gadolinium (Gd) solution using the aforementioned system, attaining a spatial resolution of approximately 10 mm. However, when reconstructing images with multiple sources (dual tubes), the signal consistency was poor due to event interference. Additionally, they employed the List Mode Maximum Likelihood Expectation Maximization (LM-MLEM) algorithm for image reconstruction, but the spatial resolution of the imaging system was still limited [17]. Based on these limitations, in 2023, the team optimized the system using the CCFIRM algorithm, integrating low-energy list-mode MLEM with polarized scattering correction. Despite this optimization, the imaging resolution, detection sensitivity, and angular resolution of the system were still relatively low [18].

The aforementioned research indicates that incorporating Compton cameras into XFCT systems can enhance imaging quality, improve spatial resolution and contrast, effectively reduce radiation dose, increase photon utilization, shorten imaging time, and improve overall imaging quality. However, in existing studies on Compton camera-based X-ray imaging, all investigations rely on imaging with a single set of Compton cameras, which presents significant limitations. Firstly, they depend on single-angle event acquisition, resulting in limited angular coverage. This limitation leads to a small number of effective events for low-concentration samples and consequently causes blurred imaging. Secondly, the angular resolution is low; the Doppler broadening of low-energy photons overlaps with single-angle errors, which reduces the spatial resolution. Thirdly, the clinical detection rate is constrained, as events from a single direction are susceptible to scattering interference. For image reconstruction, traditional MLEM algorithms suffer from slow convergence, long iteration times, sensitivity to initial values, susceptibility to local optima, poor noise resistance, high computational complexity, and demanding hardware requirements [19]. The LM-MLEM algorithm requires substantial computational resources and significant memory for processing list-mode data, is time-consuming, and produces reconstruction results prone to bias, distortion, high noise levels, and poor robustness. Concurrently, its convergence is unstable, requiring numerous iterations. When dealing with limited-angle data or insufficient data, truncation errors can occur, leading to image edge distortion [20].

This paper presents a bilateral Compton camera X-ray fluorescence imaging system. The aforementioned limitations of the unilateral structure are addressed through a symmetric layout. Full angular coverage enhances the number of effective events, providing a data foundation for imaging samples with a low concentration of 0.2%. The angular resolution (ARM FWHM) of the system is reduced to 9.35°. By incorporating the geometric properties of the system, a unique method based on geometric and angular relationships is adopted. Total Variation (TV) regularization and filtering techniques are incorporated into the traditional list-mode LM-MLEM algorithm to suppress artifact noise, and a Compton scattering back-projection optimized LM-MLEM algorithm is proposed. The use of a distance filtering mechanism in data processing enhances algorithm robustness and result quality. The iterative update process integrates normalization and auxiliary computations, avoiding local optima, balancing convergence speed and stability, significantly shortening reconstruction time, reducing computational complexity, improving image reconstruction quality and speed, and mitigating noise interference. This paper also implements three-dimensional reconstruction based on the two-dimensional results, obtaining spatial distribution information of the fluorescence source. To validate the proposed model and algorithm, simulation experiments were conducted, and the optimized two-dimensional reconstruction results were quantitatively evaluated.

## 2. Theory

### 2.1. Two-Dimensional Reconstruction Algorithm Optimization

The traditional List Mode Maximum Likelihood Expectation Maximization (LM-MLEM) algorithm is a widely used image reconstruction algorithm in fields such as medical imaging [21]. Unlike traditional projection data, list-mode data retains detailed information about each photon event, providing a more accurate reflection of the physical phenomena during the imaging process. However, it requires processing large volumes of list-mode data, and each iteration involves complex computations, making it computationally intensive, time-consuming, prone to converging to local optima, reducing edge information, and lacking noise resistance. It typically requires numerous iterations to converge to a satisfactory reconstruction, particularly for high-resolution images or large datasets. The iterative formula is given by Equation (1) [22]:

(1) $$f_{j}^{\left(n+1\right)}=\frac{f_{j}^{\left(n\right)}}{s_{j}}\sum t_{ij}\frac{1}{\sum_{k=1}^{N}t_{ik}f_{k}^{\left(n\right)}},$$

where $f_{j}^{\left(n\right)}$ is the reconstructed image intensity at voxel *j* after iteration n, $t_{ij}$ is the system matrix element linking event *i* and voxel *j*, and $S_{j}$ is the element of the sensitivity matrix for voxel *j*.

The optimization algorithm proposed in this section is specifically designed for the Compton camera X-ray fluorescence imaging system (detailed below). It introduces Total Variation (TV) regularization into the traditional LM-MLEM framework to suppress image noise and artifacts, thereby improving reconstruction quality and stability. A noise filtering mechanism is also added to eliminate invalid events. Real-time calculation of voxel contributions and dynamic construction of the system matrix are performed based on the geometric relationship between the scattering points and absorption points within the Compton camera structure. Iterative optimization incorporates normalization to balance convergence speed and stability. Furthermore, voxel threshold segmentation is employed to achieve three-dimensional reconstruction of the fluorescence source. The algorithm steps are as follows:
BEGIN Step 1: Data Collection Step 2: Hierarchical Data Preprocessing Step 3: Compton Scattering Angle Calculation and Cone Back-projection Step 4: Construction of Dynamic System Matrix and Quantification of Voxel Contribution Step 5: Iterative Optimization with TV Regularization for Voxel Intensity Update Step 6: Intermediate Result Output and Monitoring Step 7: Final Result Output END

The comparison of algorithms before and after optimization is shown in Table 1.

#### 2.1.1. Compton Scattering and Cone Back-Projection

**Figure 1:**
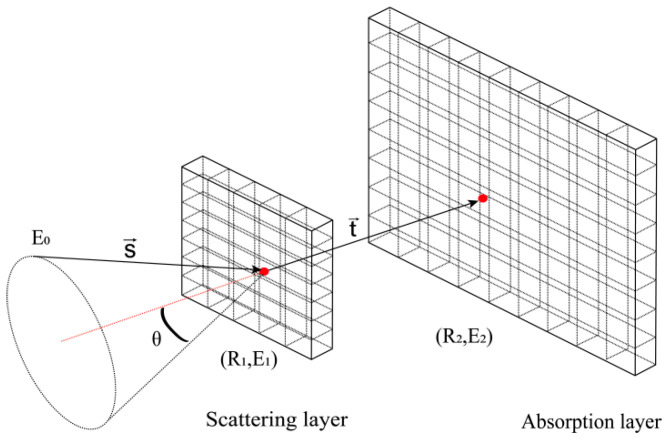
Schematic diagram of Compton camera double layer detector.

The Compton camera utilizes the Compton scattering effect to track incident photons and achieve “electronic collimation” [23]. It comprises a scattering detector and an absorption detector, as illustrated in Figure 1, and is typically used to collect photons in the energy range of tens of keV to several MeV. An incident photon collides with an outer-shell electron of an atom; the electron gains a portion of the incident photon’s energy and recoils, while the incident photon loses energy and deviates in direction. The deviation angle *θ* is termed the scattering angle. The relationship between *θ*, $E_{1}$ and $E_{2}$ is given by Equations (2) and (3):

(2) $$\theta =\arccos(1-m_{e}c^{2}(\frac{1}{E_{2}}-\frac{1}{E_{0}})),$$

(3) $$E_{0}=E_{1}+E_{2},$$

where $E_{0}$ is the initial energy of the incident photon, $m_{e}c^{2}$ is the initial electron rest energy (511 keV), $E_{1}$ and $E_{2}$ are the energy depositions at the scattering point and absorption point, respectively.

When Compton scattering occurs, the possible paths of the incident photon lie on a conical surface with the first scattering position as the vertex, the line connecting the scattering and absorption positions as the axis, and the scattering angle *θ* as the half-angle. Calculating the scattering angle using Equation (2) provides key angular information for subsequent back-projection operations.

#### 2.1.2. Dynamic System Matrix Construction

**Figure 2:**
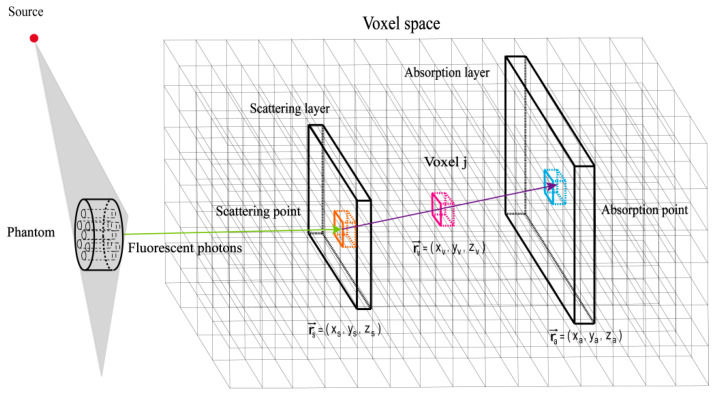
Schematic diagram of space voxel division.

The optimized algorithm employs a dynamic system matrix construction method. As shown in Figure 2, after spatial voxelization, for each valid Compton scattering event, the positions of the scattering point $\vec{r_{s}}=(x_{s},y_{s},z_{s})$ and absorption point $\vec{r_{a}}=(x_{a},y_{a},z_{a})$ are determined first. By traversing voxels and performing geometric calculations, the relationship between each spatial voxel and the scattering event is determined, specifically whether the path of the scattered photon passes through a given spatial voxel $\vec{r_{v}}=(x_{v},y_{v},z_{v})$. According to Compton scattering principles, the probability distribution function P for photons originating within a voxel to generate the observed scattering event is calculated, determining the contribution of voxels to the event and yielding the elements $a_{ij}$ of the system matrix A. The calculation method is shown in Equations (4) and (5):

(4) $$a_{ij}=\int_{V_{j}}P\cdot \mu_{j}\cdot d_{ij}dV,$$

(5) $$A=(a_{ij}),$$

where $a_{ij}$ is the system matrix element, representing the contribution of voxel j to Compton scattering event i. P represents the probability distribution function of photons passing through voxel j from the scattering point to the absorption point. $d_{ij}$ is the path length of the i-th photon within voxel j, and $\mu_{j}$ is the linear attenuation coefficient of voxel j. dV represents voxel volume element.

By calculating the relationship between events and voxels in real-time, the contribution of each valid Compton event to voxels is determined, and the system matrix is dynamically updated. Compared to traditional LM-MLEM methods, this approach not only considers photon scattering and absorption but also more accurately reflects photon propagation and energy distribution between different voxels.

#### 2.1.3. Voxel Association

This algorithm filters out noise events during the data preprocessing stage by setting a distance threshold to exclude invalid data where scattering and absorption points are too close.

Based on the geometric relationship between the scattering angle and voxel position, the information from Compton scattering events is projected into voxel space via cone back-projection. For each voxel j, its spatial position is $\vec{r_{vj}}=(x_{j},y_{j},z_{j})$, and its spatial range is defined as $\left[x_{j1},x_{j2}][y_{j1},y_{j2}][z_{j1},x_{j2}\right]$. Using the position information of the scattering and absorption points, vector operations are performed and normalized [24].

If the entry and exit points of a photon path segment through a voxel are $p_{1}$ and $p_{2}$, the voxel boundaries can be determined based on its position and size. The geometric intersection points of the photon path with the voxel are determined using vector operations, and intersecting voxels are screened using three-dimensional boundary equations. According to the Beer-Lambert law [25], the intensity attenuation and energy deposition ratio for photons traversing voxels are given by Equations (6) and (7) [26]:

(6) $$I=I_{0}e^{-\mu_{j}d_{ij}},$$

(7) $$e_{ij}=1-e^{-\mu_{j}d_{ij}},$$

where $I_{0}\;$ and I represent the energy of fluorescent photons before and after attenuation within voxel j, respectively. $d_{ij}$ represents the path length of the photon within voxel j, and $\mu_{j}$ represents the attenuation coefficient for fluorescent photons in voxel j.

If the total photon path from the source voxel to the scattering point passes through n voxels, with path lengths $d_{i1},d_{i2},\ldots ,d_{in}$, and corresponding linear attenuation coefficients $\mu_{1},\mu_{2},\ldots ,\mu_{n}$, then the probability distribution function contribution for voxel j is expressed as Equation (8):

(8) $$P_{ij}=\prod_{k=1}^{n}e^{-\mu_{k}d_{ik}},$$

Update the system matrix A according to Formulas (4), (5), and (8).

#### 2.1.4. TV Regularization Fusion Iterative Update

To address the problems of noise amplification and edge blurring in the traditional LM-MLEM iteration process, this algorithm introduces total variation (TV) regularization. TV regularization minimizes the L1 norm of the image gradient, suppressing noise while preserving edge information [27]. The L2 norm is used to measure the difference in intermediate results after iteration. The specific implementation adopts the Split Bregman algorithm, whose core idea is to decompose the non-smooth TV term into separable subproblems solved through iterative optimization [28,29]. The objective function incorporating TV regularization is given by Equation (13), and the total variation TV(f) is defined in Equations (9) and (10):

(9) $$\min_{f}\{TV(f)+\frac{1}{2\lambda_{TV}}||f-f^{\left(n+1,LM-MLEM\right)}|{|}_{2}^{2}\},$$

(10) $$TV(f)=\sum_{j}\sqrt{{\left(\nabla_{x}f_{j}\right)}^{2}+{\left(\nabla_{y}f_{j}\right)}^{2}+{\left(\nabla_{z}f_{j}\right)}^{2}},$$

where $\lambda_{TV}\;$ is the regularization strength parameter, and $f^{\left(n+1,LM-MLEM\right)}\;$ is the intermediate result of the LM-MLEM algorithm after the (n+1)-th iteration.

In this study, a finite difference approximation was used to calculate the three-dimensional gradient of voxel j, as shown in Equation (11):

(11) $$\left\{\begin{array}{l} \nabla_{x}f_{j}=f_{j+\Delta x}^{\left(n+1,LM-MLEM\right)}-f_{j}^{\left(n+1,LM-MLEM\right)}\\ \nabla_{y}f_{j}=f_{j+\Delta y}^{\left(n+1,LM-MLEM\right)}-f_{j}^{\left(n+1,LM-MLEM\right)}\\ \nabla_{z}f_{j}=f_{j+\Delta z}^{\left(n+1,LM-MLEM\right)}-f_{j}^{\left(n+1,LM-MLEM\right)} \end{array}\right.,$$

where $\nabla_{x}f_{j},\nabla_{y}f_{j},\nabla_{z}f_{j}$ are the gradients of voxel j in the x,y,z directions, respectively. When voxel j is located at the boundary of the reconstruction area, if adjacent voxels do not exist, the corresponding gradient component is set to 0; $\Delta_{x},\Delta_{y},\Delta_{z}$ are the adjacent index differences in voxels in each direction.

The Split Bregman algorithm transforms the original problem into an iterative solution by introducing dual variables $d_{x},d_{y},d_{z}$ and Bregman residuals $b_{x},b_{y},b_{z}$, suppressing gradient noise through iterative updates. The calculation method is shown in Equations (12) and (13):

(12) $$\left\{\begin{array}{l} d_{x}=\frac{\nabla_{x}f+b_{x}}{||\nabla_{x}f+b_{x}|{|}_{2}},d_{y}=\frac{\nabla_{y}f+b_{y}}{||\nabla_{y}f+b_{y}|{|}_{2}},d_{z}=\frac{\nabla_{z}f+b_{z}}{||\nabla_{z}f+b_{z}|{|}_{2}}\\ b_{x}\leftarrow b_{x}+\nabla_{x}f-d_{x},b_{y}\leftarrow b_{y}+\nabla_{y}f-d_{y},b_{z}\leftarrow b_{z}+\nabla_{z}f-d_{z} \end{array}\right.,$$

(13) $$div=\nabla \cdot (d_{x},d_{y},d_{z}),$$

where $\left||\nabla_{x}f+b_{x}||,||\nabla_{y}f+b_{y}||,||\nabla_{z}f+b_{z}|\right|$ are the L2 norms of gradients and residuals in different directions, which measure the strength of local gradients. div is the divergence of the gradient field.

By combining the regularization strength $\lambda_{TV}$ and the Bregman parameter β, the voxel values are updated using Equation (14):

(14) $$f_{j}^{\left(n+1\right)}=f_{j}^{\left(n+1,LM-MLEM\right)}+\frac{\lambda_{TV}}{\beta }\cdot div,$$

Integrating the TV regularization with the traditional LM-MLEM iteration formula yields the complete iteration formula, Equation (15):

(15) $$f_{j}^{\left(n+1\right)}=\left\{\begin{array}{l} f_{j}^{\left(n\right)}\frac{\sum_{i=1}^{m}\frac{a_{ij}y_{i}}{\sum_{k=1}^{M}a_{ik}f_{k}^{\left(n\right)}}}{\sum_{i=1}^{m}a_{ij}}\\ f_{j}^{\left(n+1,LM-MLEM\right)}+\frac{\lambda_{TV}}{\beta }\cdot div(n\geq 1) \end{array}\right.,$$

where n is the number of iterations, i is the index of the observed data, j is the index of voxels, $f_{j}^{\left(n\right)}$ is the strength of voxel j at n iterations, $f_{j}^{\left(n+1\right)}$ is the strength of voxel j at n+1 iterations, m is the upper limit of the observed data, M is the total number of voxels, $a_{ij}$ is the element of the system matrix A, and $y_{i}$ is the i-th observed data. $f_{k}^{\left(n\right)}$ is the strength of voxel j at n-th iterations.

During the iterative process, the reconstruction result matrix is output after each iteration. MATLAB is used to display the reconstruction results after each iteration for continuous assessment and refinement.

### 2.2. Three-Dimensional Reconstruction

Three-dimensional volume reconstruction is a key feature of the optimized algorithm presented in this paper. It involves converting two-dimensional projection data (or the reconstructed 2D slices) into a three-dimensional voxel model to visualize the spatial distribution and concentration of fluorescent sources [30]. This algorithm employs voxel modeling and isosurface rendering techniques [31], combined with anisotropic total variation (TV) regularization and wavelet-domain sparse constraints, to enhance reconstruction quality and detail preservation capability [32,33].

The specific steps are:
BEGIN Step 1: Data Preparation and Reading Step 2: Voxel Modeling and Data Preprocessing Step 3: Anisotropic TV Regularization Step 4: Sparse Constraint in Wavelet Domain Step 5: 3D Visualization and Rendering END

#### 2.2.1. Anisotropic TV Regularization and Wavelet Sparse Constraints

To suppress noise and artifacts in 3D reconstruction, the algorithm incorporates a dual regularization mechanism. The core of anisotropic TV regularization is gradient-based anisotropic diffusion. In smooth regions (small gradients), diffusion is enhanced to suppress noise, while in edge regions (large gradients), diffusion is weakened to preserve structural details. The formulation is shown in Equation (16):

(16) $$\frac{\partial f}{\partial t}=\lambda_{TV}\cdot \nabla \cdot (\frac{\nabla f}{|\nabla f|+\varepsilon }),$$

where $\lambda_{TV}$ is the regularization strength, ∇ is the divergence operator, ε is the diffusion coefficient, ∇f is the gradient term.

In 3D reconstruction, noise typically manifests as high-frequency components, while tissue structure corresponds to low-frequency and mid-frequency components. Sparse constraints in the wavelet domain suppress wavelet coefficients dominated by noise while preserving those dominated by the signal, achieving image denoising and feature enhancement. This algorithm implements a multi-level wavelet decomposition, thresholding, and reconstruction process. First, the two-dimensional slice array is decomposed into approximation and detail subbands. The decomposition process for one level can be expressed conceptually as Equation (17):

(17) $$f\left(x,y\right)\overset{DWT}{\to }{\left\{LL_{j},LH_{j},HL_{j},HH_{j},\right\}}_{j=1}^{J},$$

where LL is the approximation coefficient, LH is the horizontal detail, HL is the vertical detail, HH is the diagonal detail, and J is the decomposition level.

Thresholding is the core step for implementing sparse constraints. It suppresses small coefficients (likely noise) and preserves large coefficients (likely signal), making the wavelet coefficients sparse. This algorithm uses a soft threshold function, given by Equation (18):

(18) $$ST(c,T)=\left\{\begin{array}{l} \mathrm{sgn}(c)\cdot (|c|-T),|c|\geq T\\ 0,|c|<T \end{array}\right.,$$

where T is the threshold, c is the wavelet coefficient, and sgn(c) is the sign function.

The thresholded coefficients are used to reconstruct the denoised image via the inverse wavelet transform (IDWT). The specific formula is shown in Equation (19):

(19) $$f_{\mathrm{re}con}(x,y)=\sum_{j,k}c_{thresh,j,k}\psi_{j,k}^{*}(x,y),$$

where $f_{recon}(x,y)$ is the reconstructed two-dimensional image, $c_{thresh,j,k}$ are the coefficients after threshold processing, and $\Psi_{j,k}^{*}(x,y)$ is the dual wavelet function.

#### 2.2.2. Three-Dimensional Visualization Technique Based on Isosurface Rendering

**Figure 3:**
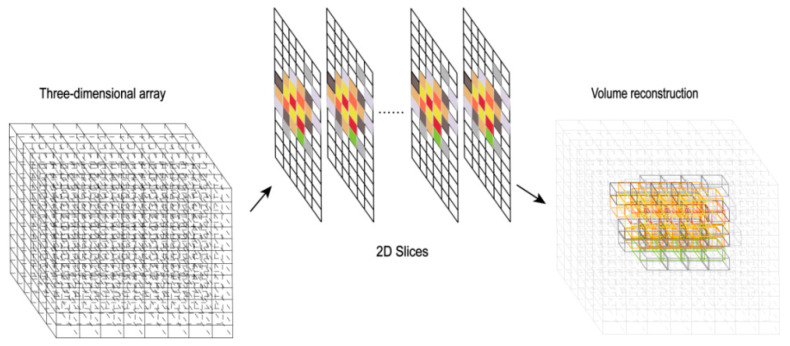
Schematic diagram of volume reconstruction.

Isosurface rendering is a key technique for converting three-dimensional volume data into intuitive two-dimensional visualizations. Based on the voxel space established in the optimized two-dimensional reconstruction algorithm, each voxel stores intensity values representing properties like energy deposition or fluorescent source concentration. The core of isosurface rendering is to extract surfaces of constant intensity value (isosurfaces) from the voxel model, converting abstract volumetric data into an intuitive 3D surface representation. Selecting an appropriate isosurface threshold is crucial to balance noise suppression and detail preservation. As shown in Figure 3, the algorithm first defines the 3D reconstruction space as a grid of voxels forming a 3D array. It then extracts 2D slices for initial processing and assigns colors. Using loop operations, isosurfaces are extracted from the 3D data. Each pixel in the rendered image corresponds to properties derived from the voxel data, with grayscale or color intensity visually representing the voxel intensity value, achieving visualization of the three-dimensional structure. The main steps include: isosurface extraction, surface normal calculation, lighting application, and rendering [34,35].

## 3. Experimental Design

### 3.1. System Construction and Parameter Design

**Figure 4:**
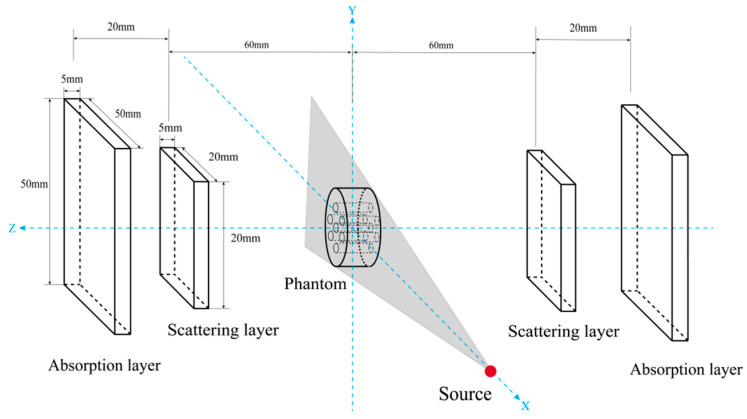
Schematic diagram of XFCT imaging system based on bilateral Compton camera detectors.

**Figure 5:**
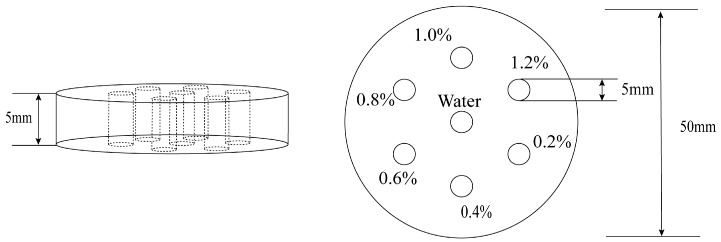
Schematic diagram of phantom.

In this study, Monte Carlo simulations were performed using Geant4 (v11.0.0). For the interaction process of low-energy X-ray fluorescent photons (60–100 keV), the G4EmPenelopePhysics list was selected [36]. This physics list exhibits significantly higher simulation accuracy for photon-matter interactions within the energy range of 1 keV to 1 MeV compared to the default physics list, and is particularly suitable for calculating the energy deposition and angular distribution of low-energy photons [37]. This study utilized the Geant4 toolkit to simulate a bilateral Compton camera X-ray fluorescence imaging system, as depicted in Figure 4. The system comprises a radiation source, a phantom, and two sets of double-layer Compton camera detectors (scattering layer and absorption layer). The scattering detector features a rectangular structure made of silicon (Si) material, with dimensions of 20 mm × 20 mm × 5 mm. The absorption detector is made of cadmium zinc telluride (CdZnTe) material, with dimensions of 50 mm × 50 mm × 5 mm, positioned behind the scattering detector to receive scattered photons. Si material exhibits high detection efficiency for low-energy photons, enabling effective capture of low-energy photons generated during Compton scattering and accurate recording of scattering position information [38]. CdZnTe material possesses efficient photon absorption capabilities, reliably absorbing scattered photons and accurately measuring their deposited energy [39]. The distance between the scattering layer and the phantom module was 60 mm, and the distance between the two detector pairs was 20 mm. The phantom module consists of a large cylinder embedded with six smaller cylinders and a central cylinder of equal size, as shown in Figure 5. The large cylinder has a radius R = 25 mm, height 5 mm, and is filled with air. Six small cylinders with radius r = 2.5 mm and height 5 mm are filled with aqueous solutions of Au nanoparticles at concentrations ranging from 0.2% to 1.2%. The central small cylinder serves as a control sample filled with deionized water. Gold (Au) was selected as the tracer element due to its high atomic number (Z = 79), high electron density (1.93 × 10^29^ electrons/m^3^), and low cytotoxicity [40,41]. The radiation source employed a conical beam, offering wider coverage compared to traditional fan-beam or parallel-beam geometries, requiring only a single scan for comprehensive data acquisition [42]. The radiation source was defined as a “gamma” source with an energy of 81 keV, slightly above the K-edge energy of gold (Au) (80.7 keV), sufficient to excite a significant number of X-ray fluorescence photons. Its position was fixed at (100 mm, 0 mm, 0 mm), oriented at a 90° angle relative to the detector plane, with the initial momentum direction randomly distributed on a disk centered at the origin. The system imaging process is as follows: First, the gamma-ray source emits gamma rays. Radiation enters the target object, interacts with it, and excites atoms to produce XRF photons. Second, the detector system registers fluorescent photons. Fluorescent photons enter the scattering layer and undergo Compton scattering. The detector in this layer records the location of Compton scattering and the energy deposition of the recoil electron. The Compton-scattered photon then travels from the scatterer and is absorbed in the second layer (absorber). This layer’s detector records the absorption position and the deposited energy.

### 3.2. Data Acquisition and Performance Indicator Analysis

After completing the geometric construction and parameter settings in Geant4, particle simulation was performed. Key data for all effective Compton events were collected using a user-defined Action class. These data encompass spatial position information and deposition energy details for the fluorescence photon emission (source), Compton scattering, and absorption processes, exported in .txt file format for subsequent analysis [43]. This study analyzed system performance metrics including detection efficiency, angular resolution, and energy resolution by varying parameters such as radiation source energy, detector geometry and size, and the concentration of high-Z elements in the sample.

#### 3.2.1. Effective Compton Event

**Figure 6:**
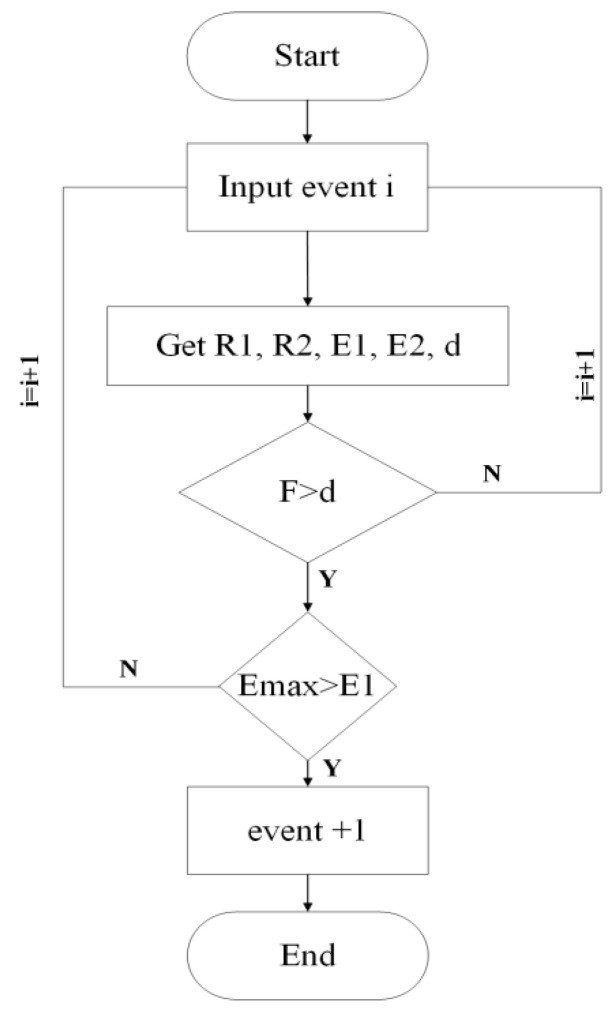
Flowchart of Effective Compton Incident Screening.

Fluorescent photons incident on the detector are considered effective detection photons if they first reach the scattering layer, undergo a Compton scattering interaction, then reach the absorption layer and are completely absorbed at a single point within it. Such events are termed effective Compton events [44,45]. The screening process for effective Compton events is illustrated in Figure 6. The overall system detection efficiency η is determined by both the scattering efficiency $\eta_{s}$ and the absorption efficiency $\eta_{a}$. Scattering efficiency refers to the probability that a fluorescent photon passing through the scattering layer undergoes Compton scattering. Absorption efficiency refers to the probability that a fluorescent photon entering the absorption layer is completely absorbed within it. The probability of an effective Compton event directly determines the system’s detection efficiency; the higher this probability, the higher the system’s detection efficiency.

#### 3.2.2. Angle Resolution and Energy Resolution

**Figure 7:**
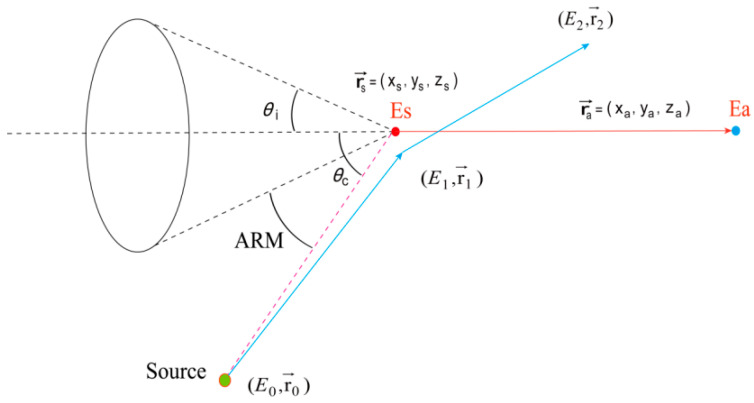
Schematic diagram of Compton event scattering angle and angular resolution.

The Angular Resolution Measure (ARM) for Compton camera imaging is defined as the angular difference between the reconstructed source direction obtained via cone back-projection and the actual source direction. This is equivalent to the angle difference between the source direction calculated based on energy deposition (using Compton kinematics) and the source direction calculated based on the positions where scattering and absorption occurred. ARM is the most critical indicator for assessing Compton camera performance [46,47,48]. As shown in Figure 7, $\theta_{i}$ represents the Compton scattering angle calculated based on the energy deposition, $\theta_{c}$ represents the cone direction angle calculated based on the positions of two points. The Compton scattering angle calculated from the detected energies is defined by Equation (20):

(20) $$\theta_{i}=\arccos(1-\frac{m_{e}c^{2}.E_{s}}{(E_{s}+E_{a}).E_{a}}),$$

where $E_{s}$ is the energy deposition at the detected scattering point, $E_{a}$ is the energy deposition at the detected absorption point, and $m_{e}c^{2}$ is initially the electron rest energy (511 keV).

The Compton scattering angle calculated based on the position of the source and the location of the Compton scattering event is defined by Equation (21):

(21) $$\theta_{c}=\arccos\left(\frac{\left(\vec{r_{s}}-\vec{r_{0}}).(\vec{r_{a}}-\vec{r_{s}}\right)}{\left|\left(\vec{r_{s}}-\vec{r_{0}}\right)\right|.\left|\left(\vec{r_{a}}-\vec{r_{s}}\right)\right|}\right),$$

where $\vec{r_{s}}$ is the position vector of the scattering point, $\vec{r_{a}}$ is the position vector of the absorption point, and $\vec{r_{0}}$ is the position vector of the source.

The Angular Resolution Measure (ARM) is then defined using Equations (20) and (21) as Equation (22):

(22) $$ARM=\theta_{i}-\theta_{c},$$

In practice, the distribution of ARM values is obtained by analyzing a large number of Compton events. The Full Width at Half Maximum (FWHM) of the ARM distribution serves as a quantitative indicator of angular resolution [49]. A smaller FWHM indicates higher angular resolution and better detection accuracy.

The energy resolution R describes the precision with which a detector can measure photon energies. Smaller energy resolution values indicate higher accuracy. In this paper, energy resolution is expressed as the ratio of the standard deviation $\sigma_{E}$ of the detector’s energy distribution for a monoenergetic source to the mean energy $\mu_{E}$, shown as a percentage in Equation (23) [50]:

(23) $$R=\frac{\sigma_{E}}{\mu_{E}}\times 100\%,$$

#### 3.2.3. Contrast-to-Noise Ratio (CNR) and Signal-to-Noise Ratio (SNR)

In evaluating the system’s image reconstruction performance, the Region of Interest (ROI) is defined as the area containing high-Z elements, with the remaining portion designated as the background area. The contrast-to-noise ratio (CNR) represents the contrast between the signal in the ROI and the background noise, while the signal-to-noise ratio (SNR) represents the strength of the signal relative to the noise within the ROI. These are two key metrics for assessing image reconstruction quality [51,52]. Higher values for both metrics indicate better signal quality and clearer images. A CNR value of 4 is a recognized threshold; values greater than or equal to 4 indicate significant contrast between the target area and the background, signifying high image quality. The specific definitions are given by Equations (24) and (25):

(24) $$CNR=\frac{\left|\mu_{ROI}-\mu_{bg}\right|}{\sigma_{bg}},$$

(25) $$SNR=\frac{\mu_{ROI}}{\sigma_{bg}},$$

where $\mu_{ROI}$ and $\mu_{bg}$ are the average signal intensity of ROI and background area, respectively, and $\sigma_{bg}$ is the standard deviation of signal intensity of background area.

## 4. Results

### 4.1. System Performance Simulation Results

**Figure 8:**
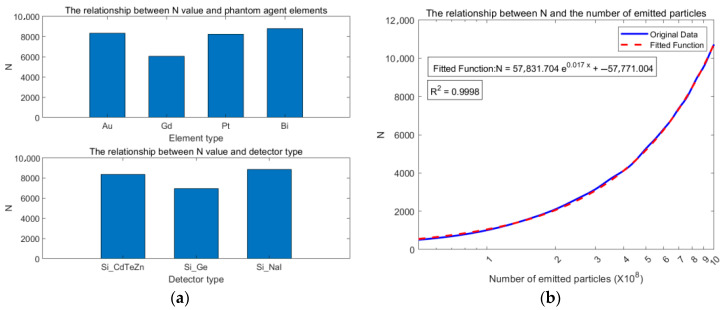
Effective Compton Event Number Variation Diagram.

This study simulated the performance of the bilateral CC-XFCT system using Geant4. The number of effective Compton events N collected by the system detector under different simulated particle numbers, phantom tracer element types, and detector material combinations was obtained. As shown in Figure 8, panel (a) displays N for different phantom tracer elements and detector types. It can be observed that, except for Gd, commonly used tracer elements like Au, Pt, and Bi allow the system to acquire sufficient effective Compton events. The Si-CdZnTe and Si-NaI detector combinations exhibit better detection efficiency than Si-Ge. Panel (b) shows the increase in N as the number of emitted source particles increases. N increases approximately linearly with the number of particles, as indicated by the fitted red curve.

**Figure 9:**
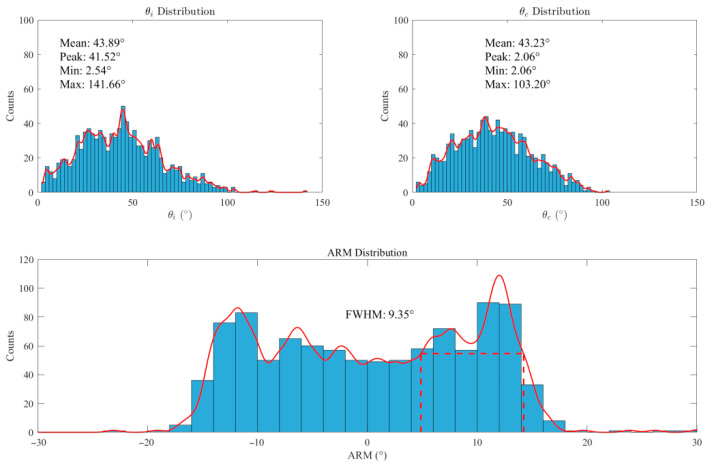
Scattering angle (°) and angular resolution (ARM) distribution. The red dashed line represents "FWHM (Full Width at Half Maximum)". FWHM refers to the distance between the two points on either side of the peak of a function where the function values are equal to half of the peak value. Here, it represents the FWHM of the ARM distribution.

In angular resolution analysis, simulations emitted 10^9^ particles using a Si-CdZnTe detector with gold (Au) as the phantom agent. Figure 9 displays the scattering angle distributions derived from energy deposition ($\theta_{i}$) and spatial coordinates ($\theta_{c}$), alongside the Angular Resolution Measure (ARM) distribution. Histograms statistically quantify the data through discrete binning, with the red curve representing the fitted theoretical distribution that smooths fluctuations and clarifies distribution trends. The mean scattering angles from both methods show close agreement. However, $\theta_{i}$ exhibits a broader angular distribution (maximum: 141.66°) compared to $\theta_{c}$ (maximum: 103.20°). The ARM distribution yields a Full Width at Half Maximum (FWHM) of 9.35°, indicating robust angular resolution. The observed multi-peak structure in the ARM profile likely arises from three factors: limited energy resolution impeding discrimination of photons with similar energies, Compton scattering multiplicity complicating angular distributions, and Doppler broadening introducing calculational errors in scattering angles.

**Figure 10:**
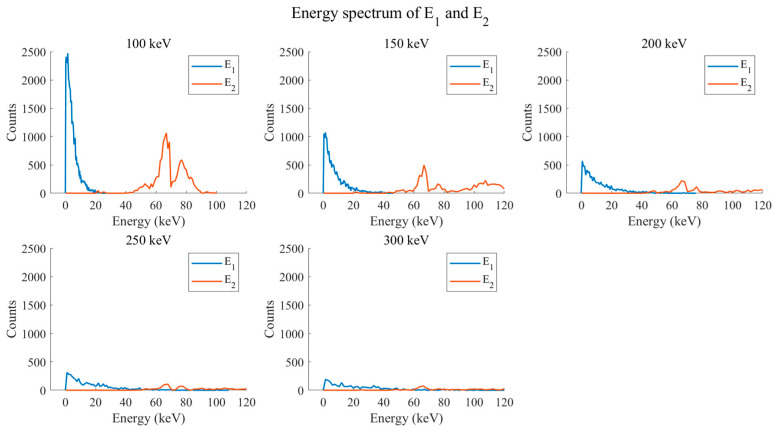
Compton camera XFCT system E1 and E2 spectra.

This study explored the energy spectra of the scattering layer detector ($E_{1}$) and absorption layer detector ($E_{2}$) under different incident photon energies (100 keV, 150 keV, 200 keV, 250 keV, 300 keV), as shown in Figure 10. The horizontal axis represents energy (keV), and the vertical axis represents counts. At various incident energies, $E_{1}$ exhibits a prominent high-count peak in the low-energy region, with counts decreasing as energy increases. $E_{2}$ shows lower counts in the low-energy region, some relatively distinct peaks in the intermediate energy region, and lower counts in the high-energy region. Although some high-energy photons not fully scattered in the scattering layer may reach the absorption layer, their number is relatively small. As incident energy increases, the absolute height of the high-count peak in $E_{1}$’s low-energy region decreases because high-energy photons are more likely to penetrate the scattering layer without undergoing low-energy scattering. The peak position and height of $E_{2}$ also change; the peak shifts towards higher energy, and its relative height decreases. This occurs because the energy distribution of photons reaching the absorption layer after high-energy incident X-ray scattering changes, and due to factors like scattering mechanisms and detector efficiency, counting rates decrease accordingly. Table 2 shows the energy resolution of the double-layer detectors at different energies. The scattering layer’s energy resolution is relatively high and decreases as the source energy increases, indicating improved energy discrimination capability at higher energies. The absorption detector’s energy resolution is lower (better) and also decreases with increasing source energy, demonstrating its inherent good energy resolution which further improves at higher energies. The trend in the number of effective Compton events N corresponds to the generation and detection of scattered photons evident in the energy spectra: more scattering events and higher detector counts occur at lower energies; fewer scattering events and lower detector counts occur at higher energies.

### 4.2. Image Reconstruction Results and Quantitative Analysis

**Figure 11:**
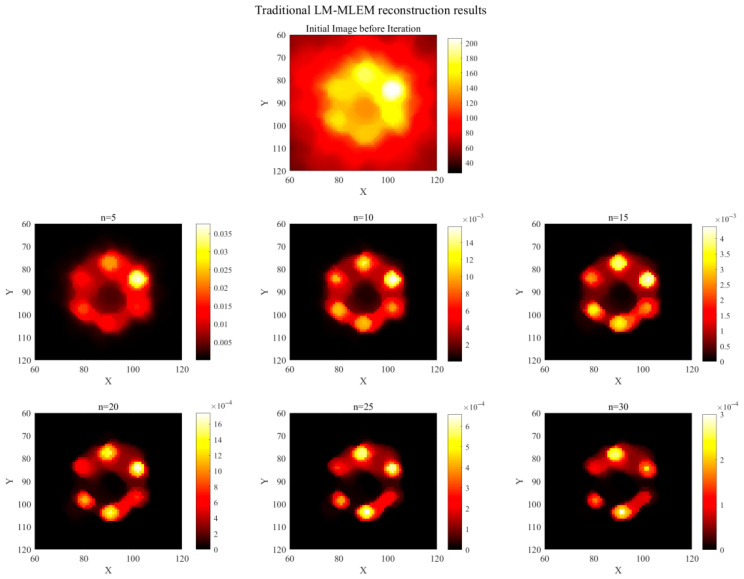
Traditional LM-MLEM algorithm reconstruction results (n = 5, 10, 15, 20, 25, 30).

**Figure 12:**
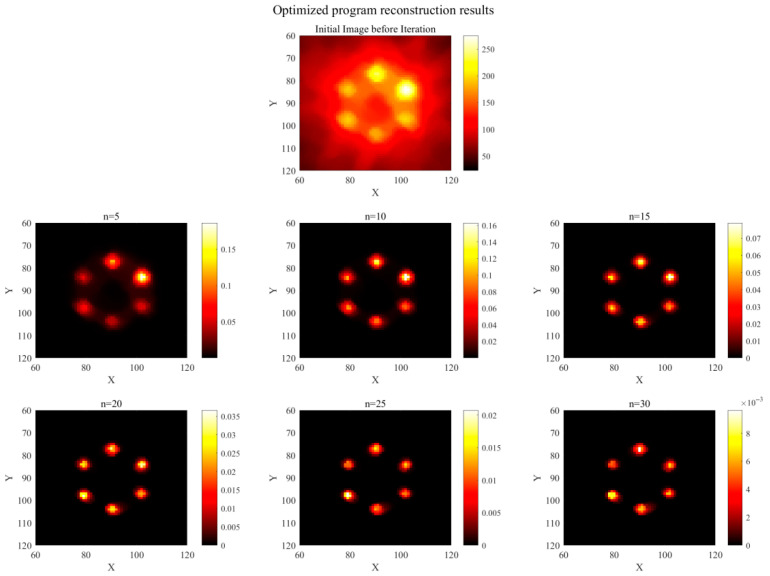
Optimization algorithm reconstruction results (n = 5, 10, 15, 20, 25, 30).

This paper proposes a list-mode MLEM volume reconstruction algorithm optimized using Compton scattering backprojection, building upon the traditional LM-MLEM algorithm. With the high-Z element in the phantom set to Au and the source energy set to 81 keV gamma rays, the simulated number of effective Compton events was obtained. Image reconstruction was performed using both algorithms, with results shown in Figure 11 (traditional) and Figure 12 (optimized). The number of iterations was 30. A comparison of the computational environment and reconstruction times is provided in Table 3.

As shown in Table 3, the traditional LM-MLEM algorithm required longer reconstruction and average iteration times. The optimized algorithm reduced the overall reconstruction time by approximately 36.5% and improved the average iteration rate by approximately 42.9%. Figure 11 shows the reconstruction results using the traditional LM-MLEM algorithm. The first row displays the initial state before iteration, with significant artifact noise and an inability to accurately identify the positions of high-Z elements. Results after 5, 10, 15, 20, 25, and 30 iterations are displayed sequentially. At n = 5, higher concentration regions are partially visible, but low-concentration regions are unclear, ROIs exhibit adhesion, and background noise is high. At n = 10, regions of different concentrations begin to appear, but noise remains significant, making precise element localization difficult. At n = 15, artifacts are reduced, but adhesion persists in low-concentration regions. With further iterations, artifact noise gradually decreases, but the spatial extent of element regions also diminishes. Significant adhesion between the two lowest concentration regions persists until n = 30, and artifacts/adhesion are not fully eliminated, indicating poor reconstruction performance. Figure 12 shows the optimized algorithm’s reconstruction results. The first row also shows significant noise in the initial state. However, compared to Figure 11, the high-Z element regions are more prominent, and their positions can be roughly determined. After different iterations, the optimized algorithm effectively reduces or eliminates artifacts and noise. At n = 5, some artifacts remain. At n = 10, artifacts are largely eliminated. At this stage, the ROIs are displayed most clearly, with the most complete spatial extent, closest resemblance to the phantom, representing the best reconstruction quality. With further iterations, artifacts disappear, but the ROI extent shrinks and edge information gradually weakens. Compared to the traditional LM-MLEM algorithm, the displayed area is significantly larger, and the lowest concentration (0.2%) is clearly visible.

**Figure 13:**
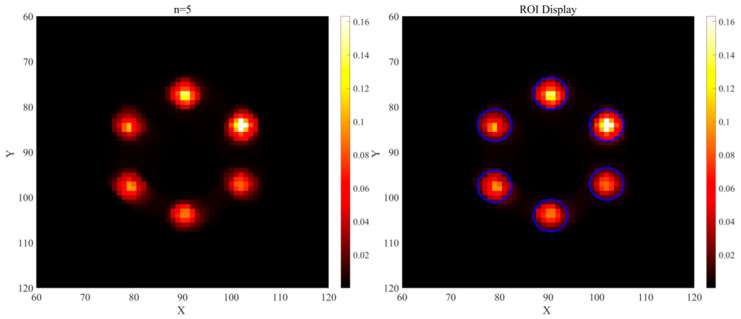
Schematic diagram of reconstructed image ROI.

**Figure 14:**
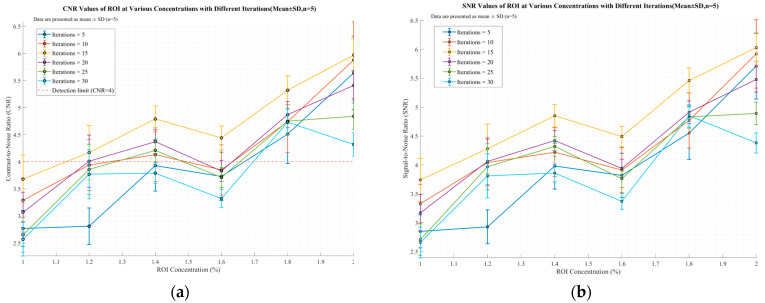
CNR and SNR values reconstructed by traditional LM-MLEM algorithm. (**a**) CNR Values of ROI at Various Concentrations with Different Iteratio (Mean ± SD, n = 5); (**b**) SNR Values of ROI at Various Concentrations with Different Iterations (Mean ± SD, n = 5)

**Figure 15:**
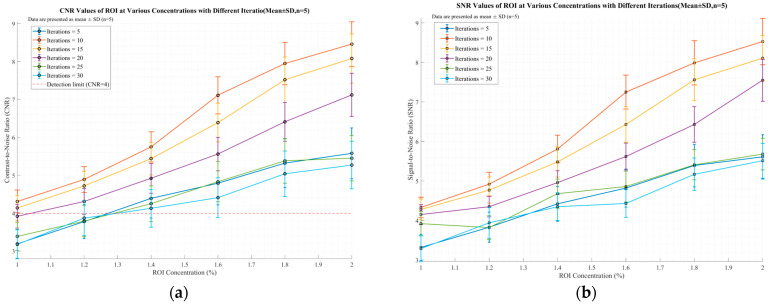
Optimize the CNR and SNR values reconstructed by the algorithm. (**a**) CNR Values of ROI at Various Concentrations with Different Iteratio (Mean ± SD, n = 5); (**b**) SNR Values of ROI at Various Concentrations with Different Iterations (Mean ± SD, n = 5).

Figure 13 illustrates the ROI selection method in the reconstructed image, where blue circles denote the regions of interest (ROI) and the remaining area is the background region. Figure 14a,b show the CNR and SNR values calculated according to Equations (24) and (25) for images reconstructed with the traditional LM-MLEM algorithm. Figure 15a,b show the calculated Contrast-to-Noise Ratio (CNR) and Signal-to-Noise Ratio (SNR) values derived from the image reconstruction results of the optimized algorithm. All data are presented as “mean ± standard deviation” after 5 independent replicate experiments. As indicated in Figure 14, when image reconstruction is performed using the traditional LM-MLEM algorithm, the overall CNR and SNR values increase with the rise in concentration under the same number of iterations; however, both values exhibit a decreasing trend when the concentration reaches 1.6%. For concentrations below 1.4%, CNR and SNR are lowest at n = 5. For concentrations above 1.4%, CNR and SNR are lowest at n = 30. Overall, CNR and SNR peak at n = 15, correlating with the best image quality for this algorithm, consistent with the visual results. According to Figure 15, when image reconstruction is performed using the optimized algorithm, the Contrast-to-Noise Ratio (CNR) and Signal-to-Noise Ratio (SNR) values exhibit an increasing trend with the rise in concentration across different numbers of iterations. Values at n = 5, 25, and 30 are relatively low overall. Both CNR and SNR peak at n = 10, and decrease with further iterations. This confirms that the optimized algorithm achieves its best reconstruction effect at n = 10.

In summary, compared to the traditional LM-MLEM algorithm, the optimized algorithm significantly improves reconstruction speed and iteration efficiency while shortening reconstruction time. The reconstruction results exhibit significantly less weakening of edge information, reduced noise interference, wider ROI spatial extent, and an optimized iteration count (only 10 iterations needed for best results), demonstrating substantial improvement.

**Figure 16:**
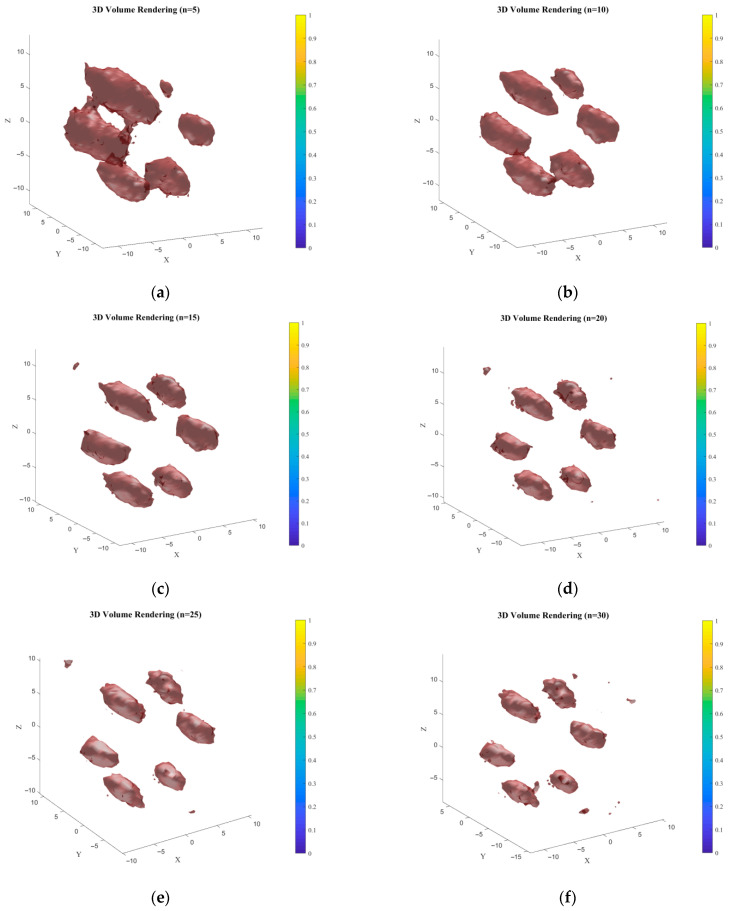
Three-dimensional reconstruction results at different iteration times. (**a**) The number of iterations is 5; (**b**) The number of iterations is 10; (**c**) The number of iterations is 15; (**d**) The number of iterations is 20; (**e**) The number of iterations is 25; (**f**) The number of iterations is 30.

**Figure 17:**
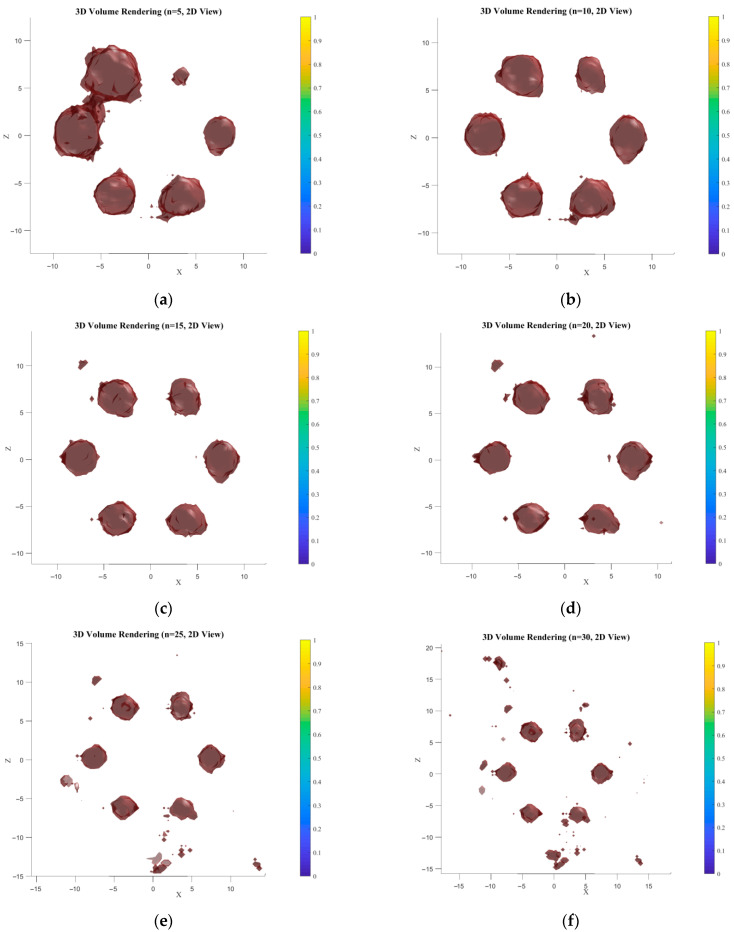
Two-dimensional slice maps corresponding to 3D reconstruction at different iteration times. (**a**) The number of iterations is 5; (**b**) The number of iterations is 10; (**c**) The number of iterations is 15; (**d**) The number of iterations is 20; (**e**) The number of iterations is 25; (**f**) The number of iterations is 30.

Based on the 3D reconstruction method of “TV Regularization-Wavelet Constraint” combined isosurface rendering proposed in the previous text, verification was conducted through multiple sets of parameter sensitivity analyses (TV regularization weights: 0.001/0.01/0.1; wavelet decomposition thresholds: 0.001/0.01/0.1; isosurface thresholds: 0.001/0.01/0.1). Finally, it was determined that the optimal 3D reconstruction results were obtained when the TV regularization weight was 0.01, the wavelet decomposition threshold was 0.01, and the isosurface threshold was 0.001. This parameter combination achieves the optimal balance between low-concentration signal capture (0.2% Au) and noise suppression, and its computational efficiency meets the clinical near-real-time requirements (approximately 1.8–2.2 h are consumed for 15–20 iterations based on the I5-13500H processor, which is 33–45% shorter than the time required for 30 iterations (3.3 h)). Figure 16 and Figure 17 show the 3D reconstruction results and corresponding XOZ plane slice plots at different iteration times. At n = 5, the results exhibit blurred features and false-positive noise, with fusion observed between adjacent structures at the target boundary, indicating incomplete convergence. The probability distribution fitting between voxels is low, and noise causes false connections. As iterations increase, the reconstruction effect gradually improves. At n = 10, the target contour becomes discernible, but structural details are still lost. High-concentration regions approximate the true sample, but low-concentration edge information is severely weakened. At n = 15, the reconstructed target regions closely resemble the sample cylinders, indicating the algorithm enters a convergence acceleration phase, with voxel probabilities approaching true values. Starting from n = 20, the shape of low-concentration regions improves towards the true value, but noise appears. For iterations beyond 20, reconstruction quality gradually degrades: target region edges become overly smooth, small details are lost, and noise increases, indicating the algorithm is trapped in local optima due to excessive iterations, and the TV regularization weight is no longer optimal. The 2D slice results in Figure 17 follow the same trend as the 3D reconstruction. In early iterations (5, 10), target boundaries in slices are blurred, shapes differ significantly, and low-concentration areas are poorly displayed (Figure 17a,b). During mid-iterations (15, 20), slices clearly present the target shape, effectively suppress noise, and closely match the true shape (Figure 17c,d). In later iterations (25, 30), slices show loss of edge details and noise recurrence in target areas (Figure 17e,f), verifying the reliability of the 3D results. In summary, under the conditions of optimal parameters and 15–20 iterations, this study can achieve high-quality 3D reconstruction of regions with a 0.2% Au concentration. Its performance is superior to that of existing unilateral Compton systems, and its computational efficiency meets the preliminary requirements for clinical near-real-time applications. However, the algorithm also has limitations: there is still a discrepancy between the edge parts of the reconstruction results and the shape of the real phantom. This discrepancy is caused by the combined effects of multiple factors, including geometric deviations in data acquisition, algorithmic dimensional coupling and regularization imbalance, and parameter settings not being adapted to the cylindrical structure. In subsequent work, efforts will be made to further reduce the imaging deviation from the real biological environment and advance clinical translation through the optimization of detector dead zones, the development of structure-adaptive algorithms, and in vivo experiments on small animals (with Au nanoparticles injected via the tail vein).

## 5. Discussion and Conclusions

This article designs a bilateral Compton camera X-ray fluorescence imaging system, which utilizes the Compton camera’s “electronic collimation” mechanism to effectively solve the problem of low photon collection rate caused by traditional X-ray fluorescence CT relying on mechanical collimation. The system is combined with Si-CdZnTe detectors to achieve an angular resolution (ARM) of 9.35° under an 81 keV radiation source. The energy resolution decreases from 10.82% to 5.65% with increasing incident energy, and the detection ability of 0.2% Au concentration is significantly better than that of a single-sided system, fully verifying the efficient detection performance of low-energy fluorescent photons. Based on this system, this article introduces TV regularization and wavelet constraints into the traditional LM-MLEM algorithm, optimizing the entire process from data preprocessing, dynamic system matrix construction, and iterative process control to result reconstruction by adapting to bilateral geometric structures, achieving two-dimensional and three-dimensional reconstruction. Through sensitivity analysis of multiple sets of parameters, it was ultimately determined that the best performance was achieved when the TV regularization weight is 0.01, the wavelet decomposition threshold is 0.01, and the isosurface threshold is 0.001. The performance improvement of the optimized algorithm is reflected in three aspects: first, efficiency. Compared with traditional LM-MLEM, the reconstruction time is shortened by 36.5%, the iteration efficiency is improved by 42.9%, and the 15–20 iteration time is controlled within 2.2 h (based on the I5-13500H processor), which is suitable for clinical near real-time preliminary requirements; The second is accuracy. In two-dimensional reconstruction, the CNR and SNR values in different concentration regions are significantly improved, and the number of iterations required to achieve the best reconstruction effect is reduced; The third aspect is stability. Three-Dimensional reconstruction experiments have shown that 15–20 iterations can balance resolution and noise, clearly presenting the 0.2% Au concentration area, verifying the algorithm’s ability to capture low concentration signals.

However, there is still a deviation between the target edge and the real phantom in the current reconstruction results. This deviation is partly due to physical limitations: firstly, the Doppler broadening effect. When low-energy photons interact with bound electrons in the detector material (Si), the thermal motion of the electrons causes inherent errors in the calculation of scattering angles, resulting in a significant broadening of the ARM distribution and ultimately affecting the edge positioning accuracy; The second is the interference of scattered photons other than fluorescent photons, which may undergo multiple scattering events. These events, when misjudged as effective events, can lead to blurred edges in low concentration areas; Thirdly, there are limitations in geometry and algorithms. The voxel event correlation of the system matrix has dimensional coupling, and the current regularization parameters are set globally, without dynamically adjusting the radial concentration gradient of the cylindrical model, further exacerbating edge deviation. In addition, this study used a uniform gold nanoparticle solution phantom, which differs from the real biological environment and needs to be further validated through phantom upgrades and in vivo experiments.

This system has demonstrated unique value in clinical translation: first, the high-sensitivity detection of 0.2% Au nanoparticles can support early detection of tumor micrometastases; second, the low radiation dose meets clinical safety requirements; and third, the bilateral layout does not require mechanical scanning, which shortens the single-slice acquisition time compared to traditional XFCT. In response to existing limitations, subsequent research will be carried out in three stages: 1. Hardware optimization: We will build a prototype Si/CdZnTe detector, reduce the detector’s dead zone, and improve the scattering probability of low-energy photons through surface coating. 2. Algorithm innovation: We will introducing GPU parallel computing and data partitioning technology to optimize the efficiency of system matrix construction and develop a “structure adaptive regularization” algorithm, combined with deep learning event filtering, to accurately eliminate multiple scattering events. 3. Physical experiment verification: We will create heterogeneous models that simulate tumor-targeted enrichment, conduct in vivo testing in mice (tail vein injection of Au nanoparticles), verify the performance of the system in real biological environments, and gradually promote clinical translation.

## Figures and Tables

**Table 1:** Comparison of algorithms before and after optimization.

Algorithm Steps	Traditional LM-MLEM	Optimized Algorithm
Data preprocessing	Collecting Compton scattered photons	Add a noise filtering mechanism to eliminate invalid events
System matrix	Fixed preset matrix	Collect effective Compton scattered photons and dynamically generate a system matrix
Iterative process	Standard MLEM iterative updates are susceptible to noise induced oscillations	Introducing Total Variation (TV) regularization to suppress image noise and artifacts
Rebuilding dimensions	2D	2D and 3D

**Table 2:** Comparison of energy resolution under different energies.

Energy (keV)	$\mathrm{Resolution}\;(E_{1}\%)$	$\mathrm{Resolution}\;(E_{2}\%)$	N
entry 1	data	data	
100	77.53	10.82	13,750
150	75.12	8.97	8323
200	74.30	7.70	6062
250	65.49	6.21	4988
300	53.06	5.65	4003

**Table 3:** Comparison of device environment and time for two reconstruction algorithms.

Algorithm Name	Traditional LM-MLEM	Optimized Algorithm
Hardware	I5-13500H processor, 16 GB, 12 cores and 16 threads, baseband of 2.5 GHz, maximum accelerated clock frequency of 4.7 GHz	
Reconstruction time (h)	5.2	3.3
Iteration time (mins)	7	4

## Data Availability

No new data were created or analyzed in this study. Data sharing is not applicable to this article.
